# Genome-wide association study and accuracy of genomic prediction for teat number in Duroc pigs using genotyping-by-sequencing

**DOI:** 10.1186/s12711-017-0311-8

**Published:** 2017-03-29

**Authors:** Cheng Tan, Zhenfang Wu, Jiangli Ren, Zhuolin Huang, Dewu Liu, Xiaoyan He, Dzianis Prakapenka, Ran Zhang, Ning Li, Yang Da, Xiaoxiang Hu

**Affiliations:** 1 State Key Laboratory for Agrobiotechnology, China Agricultural University, Beijing, 100193 China; 2 Department of Animal Science, University of Minnesota, Saint Paul, MN 55108 USA; 3 National Engineering Research Center for Breeding Swine Industry, South China Agricultural University, Guangdong, 510642 China

## Abstract

**Background:**

The number of teats in pigs is related to a sow’s ability to rear piglets to weaning age. Several studies have identified genes and genomic regions that affect teat number in swine but few common results were reported. The objective of this study was to identify genetic factors that affect teat number in pigs, evaluate the accuracy of genomic prediction, and evaluate the contribution of significant genes and genomic regions to genomic broad-sense heritability and prediction accuracy using 41,108 autosomal single nucleotide polymorphisms (SNPs) from genotyping-by-sequencing on 2936 Duroc boars.

**Results:**

Narrow-sense heritability and dominance heritability of teat number estimated by genomic restricted maximum likelihood were 0.365 ± 0.030 and 0.035 ± 0.019, respectively. The accuracy of genomic predictions, calculated as the average correlation between the genomic best linear unbiased prediction and phenotype in a tenfold validation study, was 0.437 ± 0.064 for the model with additive and dominance effects and 0.435 ± 0.064 for the model with additive effects only. Genome-wide association studies (GWAS) using three methods of analysis identified 85 significant SNP effects for teat number on chromosomes 1, 6, 7, 10, 11, 12 and 14. The region between 102.9 and 106.0 Mb on chromosome 7, which was reported in several studies, had the most significant SNP effects in or near the *PTGR2*, *FAM161B*, *LIN52*, *VRTN*, *FCF1*, *AREL1* and *LRRC74A* genes. This region accounted for 10.0% of the genomic additive heritability and 8.0% of the accuracy of prediction. The second most significant chromosome region not reported by previous GWAS was the region between 77.7 and 79.7 Mb on chromosome 11, where SNPs in the *FGF14* gene had the most significant effect and accounted for 5.1% of the genomic additive heritability and 5.2% of the accuracy of prediction. The 85 significant SNPs accounted for 28.5 to 28.8% of the genomic additive heritability and 35.8 to 36.8% of the accuracy of prediction.

**Conclusions:**

The three methods used for the GWAS identified 85 significant SNPs with additive effects on teat number, including SNPs in a previously reported chromosomal region and SNPs in novel chromosomal regions. Most significant SNPs with larger estimated effects also had larger contributions to the total genomic heritability and accuracy of prediction than other SNPs.

**Electronic supplementary material:**

The online version of this article (doi:10.1186/s12711-017-0311-8) contains supplementary material, which is available to authorized users.

## Background

A sufficient number of teats is necessary for a sow to rear its piglets to weaning age. Many putative QTL (quantitative trait loci) for teat number have been reported on most of the porcine chromosomes, but most of these were detected using microsatellite markers and lacked specific gene targets [1]. Genome-wide association studies (GWAS) using single nucleotide polymorphisms (SNPs) and analyses of candidate genes have identified several specific gene targets that affect teat number in swine. A GWAS using 42,654 SNPs on 936 Large White pigs reported 39 QTL with 211 significant SNP effects on teat number [2]. Among those SNP effects, the region between 102.0 and 105.2 Mb on chromosome 7 had the most significant effects and the percentage of the genetic variance explained by SNPs in this region ranged from 0.04 to 2.51%. Within this region, the *VRTN* and *PROX2* genes were identified as the most convincing candidate genes. The chromosomal locations of the significant SNPs that were detected in this GWAS differed from all previously reported QTL for teat number that have been compiled in the animal QTL database [1]. Another GWAS, using 32,911 SNPs on 1550 Large White pigs, reported 21 QTL with additive effects on chromosomes 6, 7 and 12, one QTL with a dominant effect on chromosome 4, and identified *VRTN* as the most promising candidate gene for teat number [3]. A third GWAS using 41,647 SNPs on 1657 Large White pigs found 65 significant SNPs on chromosomes 1, 2, 7, 8, 12 and 14, including SNPs in the region 102.9 between 105.2 Mb on chromosome 7 [4]. A fourth GWAS using 39,778 SNPs identified the *VRTN* gene with pleiotropic and desirable effects on thoracic vertebral number, teat number and carcass (body) length across four pig populations, and showed that, of all SNPs on chromosome 7, a SNP within the *VRTN* gene had the most significant effect on teat number in Duroc pigs [5]. Among all significant SNPs that have been detected for teat number by GWAS, the significance of the *VRTN* gene on chromosome 7 achieved the widest consensus and has been identified as a strong candidate gene for teat number [2–5]. However, in the literature some discrepancies regarding the most significant location and many SNP effects in other genomic regions have been reported. A GWAS using the porcine 60 K SNP chip on a F2 population from a cross between Landrace and Korean pigs identified highly significant SNPs on chromosome 7 that were more than 40 Mb away from the *VRTN* gene [6], and in another GWAS using 36,588 SNPs and 1024 Duroc pigs, the most significant SNPs on chromosome 7 were found 2 to 3 Mb downstream of the *VRTN* region [7]. However, other than for the *VRTN* region, there is little consensus among the GWAS results on genomic regions that affect teat number [2–4, 6, 7]. Therefore, additional studies are needed to identify the genetic factors that affect swine teat number. Furthermore, it is unclear what the impact of the highly significant SNPs is on the accuracy of genomic prediction for teat number.

The objective of this study was to identify genetic factors that affect teat number in pigs, evaluate the accuracy of genomic prediction, and evaluate the contribution of significant genes and genomic regions to the heritability and accuracy of genomic prediction using 41,108 autosomal SNPs from genotyping-by-sequencing (GBS) on 2936 Duroc boars.

## Methods

### Animals, phenotyping, and genotyping-by-sequencing

**Fig. 1:**
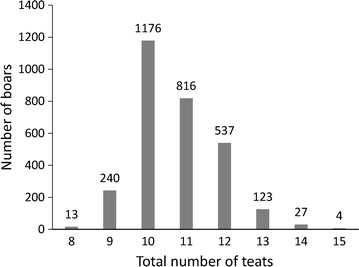
Phenotypic distribution of total teat number in Duroc boars (N = 2936)

Animal and phenotype data used for this study were provided by Guangdong Wen’s Foodstuff Group (Guangdong, China). The study population included 2936 Duroc boars born from September 2011 to September 2013 in 1456 litters from 79 sires with one to three piglets per litter, and all pigs were managed at a single nucleus farm. The left and right teats were counted separately within 48 h after birth and only normal teats were recorded. In this study, the phenotype used for ‘teat number’ was the total number of teats that was equal to the sum of the left and right normal teats. The ‘mean ± (standard deviation)’ of teat number was 10.72 ± 1.72. The phenotypic values followed a near bell-shaped distribution (Fig. 1), which was similar to the leptokurtic distribution of teat number that is observed for Landrace and Large White pigs [8], with most animals (1992 out of 2936) having 10 or 11 teats.

Genomic DNA was extracted from ear tissue of all 2936 Duroc boars and quantified using a Qubit 2.0 Fluorometer. DNA concentrations were normalized to 50 ng/ml in 96-well plates. A two-enzyme i.e. *EcoR*I and *Msp*I genotyping-by-sequencing (GBS) was used. A set of 96 forward barcoded adapters with an *EcoR*I overhang were designed by the GBS Barcode Generator (http://www.deenabio.com/services/gbs-adapters), and the reverse adapter with a *Msp*I overhang was designed according to [9]. DNA samples (150 ng each) were digested with *EcoR*I and *Msp*I, then ligated to the designed adapters. Following adapter ligation, samples were pooled in 96-plex and size-selected using two cycles of purification with Agencourt AMPure XP Beads (Beckman Coulter, Pasadena, CA). The purified libraries were amplified by PCR and then sequenced on Illumina NextSeq500 by a 90-bp single-end sequencing. SNP genotypes were called according to the pipeline implemented in Tassel 5.0 with default parameters [10], and Beagle 4.0 was used to impute missing SNP genotypes. A total of 90,051 SNPs were identified for the population used in this study. SNP filtering was based on the following criteria: only SNPs that had a minor allele frequency higher than 5%, for which the frequency of the least frequent homozygous genotype was at least 0.01, and passed the Hardy–Weinberg equilibrium test (p ≥ 10^−6^) were retained. Among the autosomal SNPs, 41,108 SNPs satisfied these requirements and were used for analyses.

### GWAS analysis

**Fig. 2:**
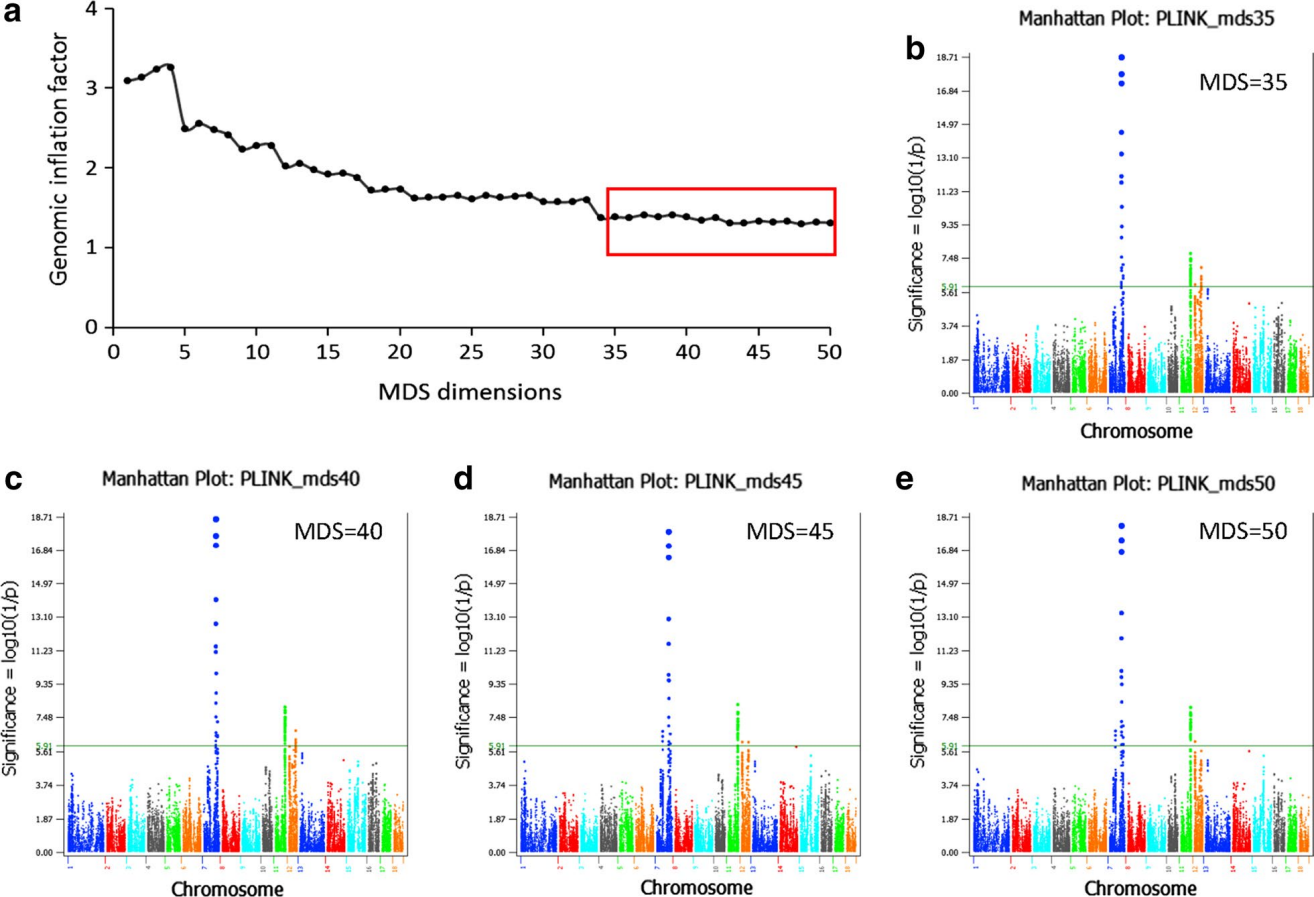
Effect of the multidimensional scaling (MDS) dimensions on the genomic inflation factor and on Manhattan plots of SNP significance. **a** Genomic inflation factor remained relatively unchanged as the number of MDS dimensions increased beyond the first 35 dimensions. **b**–**e** GWAS significance from PLINK using the first 35 to 50 MDS dimensions, showing that the significance patterns were virtually unchanged, with the exception of those for chromosome 12, which displayed decreasing significance as the number of MDS dimensions increased. All p values in the figures are on the log(1/p) scale

Three single-SNP methods were used for the GWAS analysis: a *t* test of additive and dominance SNP effects using a generalized least squares (GLS) analysis that takes intraclass correlation of sibs into account and is implemented in the EPISNP2 program [11, 12], and the least squares (LS) analyses of additive effects by PLINK [13] and EPISNP1 [11] with population stratification correction using the first 50 dimensions from multidimensional scaling (MDS) as covariates. We report the PLINK and EPISNP1 results using the first 35 MDS dimensions for stratification correction because the genomic inflation factor [14] and the patterns of Manhattan plots of SNP significance stabilized when fitting the first 35 MDS dimensions (Fig. 2).

The statistical model for the EPISNP2 analysis was:

$${\mathbf{y}} = {\mathbf{X}}_{\text{b}} {\mathbf{b}} + {\mathbf{Xg}} + {\mathbf{Zf}} + {\mathbf{e}},$$

where ${\mathbf{y}}$ is the vector of phenotypic values, ${\mathbf{b}}$ is the vector of fixed year-month effects, ${\mathbf{X}}_{\text{b}}$ is the incidence matrix for ${\mathbf{b}}$, ${\mathbf{g}}$ is the vector of the effects of SNP genotypes, ${\mathbf{X}}$ is the incidence matrix of ${\mathbf{g}}$, ${\mathbf{f}}$ is the vector of random family effects with a common variance $\upsigma_{\text{f}}^{2}$ for sibs in the same family, and ${\mathbf{Z}}$ is the incidence matrix of ${\mathbf{f}}$. The variance–covariance matrix of the family effects was assumed to be ${\mathbf{G}} = {\text{Var}}\left( {\mathbf{f}} \right) = {\mathbf{I}}\upsigma_{\text{f}}^{2}$, where ${\mathbf{I}}$ is an identity matrix, and the phenotypic variance–covariance matrix is ${\text{Var}}\left( {\mathbf{y}} \right) = {\mathbf{V}} = {\mathbf{ZGZ}}^{\prime } + {\mathbf{I}}\upsigma_{\text{e}}^{2}$ [12].

The statistical model for the PLINK analysis was:

$${\mathbf{y}} = {\mathbf{X}}_{\text{b}} {\mathbf{b}} + {\mathbf{X}}_{1} {\mathbf{b}}_{1} +\upalpha{\mathbf{x}} + {\mathbf{e}},$$

where ${\mathbf{b}}_{1}$ is the vector of fixed effect(s) of the MDS dimension(s), ${\mathbf{X}}_{1}$ is the matrix of the MDS dimension(s) as calculated by PLINK from the SNP matrix of identity-by-state [13], α is the additive SNP effect, and ${\mathbf{x}}$ is a column vector of genotype codes for α created by PLINK.

The statistical model for EPISNP1 analysis was:

$${\mathbf{y}} = {\mathbf{X}}_{\text{b}} {\mathbf{b}} + {\mathbf{X}}_{1} {\mathbf{b}}_{1} + {\mathbf{Xg}} + {\mathbf{e}},$$

where the matrices have the same definitions as in the previous two models. Significance tests for additive and dominance SNP effects by EPISNP1 and EPISNP2 were implemented by *t* tests for the additive and dominance contrasts of the estimated SNP genotypic values [11, 12, 15].

### Genomic heritability and accuracy of genomic prediction

Genomic heritability and genomic prediction were estimated by using a mixed model with additive and dominance effects as described previously [16–18]. Briefly, the mixed model for heritability estimation and genomic prediction was:

$${\mathbf{y}} = {\mathbf{X}}_{\text{b}} {\mathbf{b}} + {\mathbf{Za}} + {\mathbf{Zd}} + {\mathbf{e}},$$

with ${\text{Var}}\left( {\mathbf{y}} \right) = {\mathbf{V}} = {\mathbf{ZA}}_{\text{g}} {\mathbf{Z}}^{\prime }\upsigma_{\upalpha}^{2} + {\mathbf{ZD}}_{\text{g}} {\mathbf{Z}}^{\prime }\upsigma_{\updelta}^{2} + {\mathbf{I}}\upsigma_{\text{e}}^{2}$, where ${\mathbf{Z}}$ is an incidence matrix allocating phenotypic observations to each individual, ${\mathbf{a}}$ is the vector of genomic additive (breeding) values, ${\mathbf{d}}$ is the vector of genomic dominance values or dominance deviations, ${\mathbf{A}}_{\text{g}}$ is a genomic additive relationship matrix calculated from the SNPs, ${\mathbf{D}}_{\text{g}}$ is a genomic dominance relationship matrix calculated from the SNPs, $\upsigma_{\upalpha}^{2}$ is the additive variance, $\upsigma_{\updelta}^{2}$ is the dominance variance, and $\upsigma_{\text{e}}^{2}$ is the residual variance. The ${\mathbf{A}}_{\text{g}}$ and ${\mathbf{D}}_{\text{g}}$ matrices were calculated using Definition II of genomic relationships implemented by the GVCBLUP package, and variance components of additive, dominance and random residual values were estimated by genomic restricted maximum likelihood estimation (GREML) using the GREML_CE program in the GVCBLUP package [18]. The genomic heritability was defined as: ${\text{h}}_{\upalpha}^{2} =\upsigma_{\upalpha}^{2} /\upsigma_{\text{y}}^{2}$, i.e. the narrow-sense heritability, ${\text{h}}_{\updelta}^{2} =\upsigma_{\updelta}^{2} /\upsigma_{\text{y}}^{2}$, i.e. the dominance heritability, and ${\text{h}}_{\text{t}}^{2} = {\text{h}}_{\upalpha}^{2} /{\text{h}}_{\updelta}^{2}$, i.e. the broad-sense heritability, where $\upsigma_{\text{y}}^{2} =\upsigma_{\upalpha}^{2} +\upsigma_{\updelta}^{2} +\upsigma_{\text{e}}^{2}$ is the phenotypic variance. The genomic best linear unbiased prediction (GBLUP) of additive, dominance and genetic values of individuals in the training and validation samples were calculated at the last iteration of the GREML.

A tenfold validation study was conducted to evaluate the prediction accuracy. The 2936 Duroc boars were randomly divided into 10 validation datasets of 293 individuals except the 10th sample, which included 299 individuals. For each of the 10 validation analyses, phenotypic observations in the validation dataset were omitted in the GBLUP calculation. Three measures of prediction accuracy were calculated and compared: $\widehat{\text{R}}_{{0{\text{jp}}}} = {\text{corr}}\left( {\widehat{\text{g}}_{{0{\text{j}}}} ,\;{\text{y}}_{0} } \right)$, which is the observed accuracy of predicting the phenotypic values in the validation population and is calculated as the correlation between the estimated genetic values ($\widehat{\text{g}}_{{0{\text{j}}}}$) and the phenotypic observations (${\text{y}}_{0}$) of validation individuals, averaged across all validation datasets; ${\text{R}}_{{0{\text{j}}}} = {\text{corr}}\left( {\widehat{\text{g}}_{{0{\text{j}}}} ,\;{\text{g}}_{{0{\text{j}}}} } \right)$, which is the expected accuracy of predicting the true genetic values (${\text{g}}_{{0{\text{j}}}}$) of individuals in the validation population and is calculated as the square root of the reliability estimate for each individual from the GVCBLUP package [18], where ‘${\text{j}} =\upalpha$’ indicates additive prediction, ‘${\text{j}} =\updelta$’ indicates dominance prediction, and ‘${\text{j}} = {\text{t}}$’ indicates prediction of total genetic value; and ${\text{R}}_{{0{\text{jp}}}} = {\text{R}}_{{0{\text{j}}}} \sqrt {{\text{h}}_{\text{j}}^{2} }$, which is the expected accuracy of predicting the phenotypic values, where ${\text{h}}_{\text{j}}^{2}$ is the genomic narrow-sense (${\text{j}} =\upalpha$), dominance (${\text{j}} =\updelta$), or broad-sense (${\text{j}} = {\text{t}}$) heritability. The accuracy of predicting phenotypic values was previously termed as ‘predictive ability’ [19] to distinguish it from ‘expected prediction accuracy’ of predicting genetic values (${\text{R}}_{{0{\text{j}}}}$ in this study). The formula of the expected accuracy of predicting phenotypic values, ${\text{R}}_{{0{\text{jp}}}} = {\text{R}}_{{0{\text{j}}}} \sqrt {{\text{h}}_{\text{j}}^{2} }$, is a slightly different form of the relationship between ‘predictive ability’ and ‘prediction accuracy’ [19]. The mathematical difference between ${\text{R}}_{{0{\text{jp}}}}$ and ${\text{R}}_{{0{\text{j}}}}$ is in the denominators of these two measures: the denominator of ${\text{R}}_{{0{\text{jp}}}}$ is the phenotypic standard deviation, whereas the denominator of ${\text{R}}_{{0{\text{j}}}}$ is the genetic standard deviation, which is necessarily smaller than the phenotypic standard deviation in the presence of non-zero residual variance. Therefore, ${\text{R}}_{{0{\text{j}}}}$ is the upper limit of ${\text{R}}_{{0{\text{jp}}}}$ but this upper limit may not hold for the observed ${\text{accuracy}}$ of predicting phenotypic values ($\widehat{\text{R}}_{{0{\text{jp}}}}$) due to unknown variations in the data or genetic mechanisms that are not explained by the statistical model. The observed accuracy of predicting genetic values can only be defined when the true genetic values such as simulated genetic values are known [16] but they could not be defined in this study because the true genetic values were unknown.

### Contribution of significant SNPs and genomic regions to total heritability and prediction accuracy

The contributions of each SNP to additive, dominance and total heritability can be estimated [18]. However, as shown by the results of this study, the contribution of each SNP is affected by the number of SNPs in the mixed model: the larger is the number of SNPs, the smaller is the contribution of each SNP. To avoid this dependency in the mixed model for GREML and to estimate each SNP’s independent contribution to genomic heritability and prediction accuracy, we used the approach of ‘partial heritability’ and ‘partial accuracy’ based on differences in heritability and prediction accuracy between a full model and a reduced model. The full model fits all SNPs as random effects and the reduced model fits the target SNP or SNPs as fixed effects to remove their effects from the phenotypic values. The reduced model was as follows:

$${\mathbf{y}} = {\mathbf{X}}_{\text{f}} {\mathbf{b}} + {\mathbf{Xs}} + {\mathbf{Za}} + {\mathbf{Zd}} + {\mathbf{e}},$$

where ${\mathbf{s}}$ is a column vector of fixed SNP effects and ${\mathbf{X}}$ is the incidence matrix of ${\mathbf{s}}$. The phenotypic variance–covariance matrix was assumed to be the same as in the full model, i.e., ${\text{Var}}\left( {\mathbf{y}} \right) = {\mathbf{V}} = {\mathbf{ZA}}_{\text{g}} {\mathbf{Z}}^{\prime }\upsigma_{\upalpha}^{2} + {\mathbf{ZD}}_{\text{g}} {\mathbf{Z}}^{\prime }\upsigma_{\updelta}^{2} + {\mathbf{I}}\upsigma_{\text{e}}^{2}$, but variance components were estimated under the reduced model. Let $\widehat{\text{h}}^{2}$ $(\widehat{\text{h}}_{\text{i}}^{2} )$ be the estimated heritability from the full (reduced) model, and ${\text{R}}_{0}$ $\left( {{\text{R}}_{{0{\text{i}}}} } \right)$ a measure of prediction accuracy for the full (reduced) model. Then, the relative contribution of the $i$th SNP or the $i$th set of SNPs to the total heritability was calculated as ${\text{c}}_{\text{hi}}^{2} = 1 - \widehat{\text{h}}_{\text{i}}^{2} /\widehat{\text{h}}^{2}$, and the relative contribution of the $i$th SNP or the $i$th set of SNPs to the prediction accuracy as ${\text{c}}_{\text{ri}} = 1 - {\text{R}}_{{0{\text{i}}}} /{\text{R}}_{0}$.

## Results

### Genomic heritability and prediction accuracy

The estimate of genomic narrow-sense heritability ($\widehat{\text{h}}_{\upalpha}^{2}$) was 0.365 ± 0.030, of dominance heritability ($\widehat{\text{h}}_{\updelta}^{2}$) was 0.035 ± 0.019, of broad-sense heritability (${\text{h}}_{\text{t}}^{2}$) was 0.400 ± 0.034, and the estimate of narrow-sense heritability for the mixed model with additive effects only, was 0.368 ± 0.030, which is slightly higher than the corresponding estimate for the mixed model with additive and dominance effects (Table 1). The observed accuracy of predicting phenotypic values from the tenfold validation study was 0.437 ± 0.064 for the mixed model with additive and dominance SNP effects $(\widehat{\text{R}}_{{0{\text{tp}}}}$, Model 1A in Table 2), and was 0.435 ± 0.064 for the mixed model with additive effects only $(\widehat{\text{R}}_{{0\upalpha{\text{p}}}}$, Model 2A in Table 2), which is only 0.46% lower than that from the mixed model with additive and dominance effects. These slight differences in both heritability and accuracy of prediction between the additive model and the model with additive and dominance effects indicates that additive SNP effects were the primary genetic effects that affect teat number and that dominance SNP effects only had a negligible contribution to the prediction accuracy for teat number.

**Table 1:** Estimates of genomic heritabilities for teat number using 41,108 autosomal SNPs on 2936 Duroc boars

Model	All SNPs as random effects	85 significant SNPs removed	85 significant SNPs as fixed effects
Additive and dominance effects	$\widehat{\text{h}}_{\upalpha}^{2} = 0.365 \pm 0.030$	$\widehat{\text{h}}_{\upalpha}^{2} = 0.346 \pm 0.030$ $- {\text{c}}_{\text{hi}}^{2} = \, - 5.20\%$	$\widehat{\text{h}}_{\upalpha}^{2} = \, 0.260 \pm 0.030$ $- {\text{c}}_{\text{hi}}^{2} = \, - 28.77\%$
	$\widehat{\text{h}}_{\updelta}^{2} = 0.035 \pm 0.019$	$\widehat{\text{h}}_{\updelta}^{2} = 0.036 \pm 0.020$ $- {\text{c}}_{\text{hi}}^{2} = \, + 2.86\%$	$\widehat{\text{h}}_{\updelta}^{2} = \, 0.037 \pm 0.022$ $- {\text{c}}_{\text{hi}}^{2} = \, + 5.71\%$
	$\widehat{\text{h}}_{\text{t}}^{2} = 0.400 \pm 0.034$	$\widehat{\text{h}}_{\text{t}}^{2} = 0.382 \pm 0.034$ $- {\text{c}}_{\text{hi}}^{2} = \, - 4.50\%$	$\widehat{\text{h}}_{\text{t}}^{2} = \, 0.297 \pm 0.036$ $- {\text{c}}_{\text{hi}}^{2} = \, - 25.75\%$
Additive effects only	$\widehat{\text{h}}_{\upalpha}^{2} = \, 0.368 \pm 0.030$	$\widehat{\text{h}}_{\upalpha}^{2} = 0.350 \pm 0.030$ $- {\text{c}}_{\text{hi}}^{2} = \, - 4.89\%$	$\widehat{\text{h}}_{\upalpha}^{2} = \, 0.263 \pm 0.030$ $- {\text{c}}_{\text{hi}}^{2} = \, - 28.53\%$

${\text{h}}_{\upalpha}^{2}$ = narrow-sense heritability. ${\text{h}}_{\updelta}^{2}$ = dominance heritability. ${\text{h}}_{\text{t}}^{2}$ = broad-sense heritability = ${\text{h}}_{\upalpha}^{2} + {\text{h}}_{\updelta}^{2}$. $- {\text{c}}_{\text{hi}}^{2}$ = decrease in heritability relative to the heritability estimated by using all SNPs fitted as random effects

**Table 2:** Accuracies of genomic prediction for the phenotypic values and true genetic values of teat number using 41,108 autosomal SNPs on 2936 Duroc boars in a tenfold validation study

Model and accuracy change	$\widehat{\text{R}}_{{0{\text{tp}}}} = {\text{corr}}\left( {\widehat{\text{g}}_{{0{\text{j}}}} ,{\text{y}}_{0} } \right)$	${\text{R}}_{{0{\text{jp}}}} = {\text{R}}_{{0{\text{j}}}} \sqrt {{\text{h}}_{\text{j}}^{2} }$	${\text{R}}_{{0{\text{j}}}} = {\text{corr}}\left( {\widehat{\text{g}}_{{0{\text{j}}}} ,{\text{g}}_{{0{\text{j}}}} } \right)$
Model 1A	$\widehat{\text{R}}_{{0{\text{tp}}}}$ = 0.437 ± 0.064	${\text{R}}_{{0{\text{tp}}}}$ = 0.460	${\text{R}}_{{0{\text{t}}}}$ = 0.728 ± 0.004
Model 1B	$\widehat{\text{R}}_{{0{\text{tp}}}}$ = 0.279 ± 0.076	${\text{R}}_{{0{\text{tp}}}}$ = 0.360	${\text{R}}_{{0{\text{t}}}}$ = 0.661 ± 0.007
$- {\text{c}}_{\text{ri}}$ of 1B relative to 1A	−36.16%	−21.74%	−9.20%
Model 2A	$\widehat{\text{R}}_{{0\upalpha{\text{p}}}}$ = 0.435 ± 0.064	${\text{R}}_{{0\upalpha{\text{p}}}}$ = 0.425	${\text{R}}_{{0\upalpha}}$ = 0.700 ± 0.007
Model 2B	$\widehat{\text{R}}_{{0\upalpha{\text{p}}}}$ = 0.275 ± 0.074	${\text{R}}_{{0\upalpha{\text{p}}}}$ = 0.320	${\text{R}}_{{0\upalpha}}$ = 0.624 ± 0.009
$- {\text{c}}_{\text{ri}}$ of 2B relative to 2A	−36.78%	−24.70%	−10.86%
Model 3A	$\widehat{\text{R}}_{{0{\text{tp}}}}$ = 0.426 ± 0.066	${\text{R}}_{{0{\text{tp}}}} = \, 0.446$	${\text{R}}_{{0{\text{t}}}}$ = 0.721 ± 0.004
$- {\text{c}}_{\text{ri}}$ of 3A relative to 1A	−2.52%	−3.04%	−0.96%
Model 4A	$\widehat{\text{R}}_{{0\upalpha{\text{p}}}} = 0.424 \pm 0.066$	${\text{R}}_{{0\upalpha{\text{p}}}} = \, 0.409$	${\text{R}}_{{0\upalpha}} = 0.691 \pm 0.007$
$- {\text{c}}_{\text{ri}}$ of 4A relative to 2A	−2.53%	−3.76%	−1.28%

Model 1A has additive and dominance effects and uses all 41,108 autosome SNPs. Model 1B is a modification of Model 1A by using the 85 significant SNPs as fixed non-genetic effects. Model 2A has additive effects only and uses all 41,108 autosome SNPs. Model 2B is a modification of Model 2A by using the 85 significant SNPs as fixed non-genetic effects. Model 3A has additive and dominance effects and uses 41,023 autosomal SNPs after removing the 85 significant SNPs. Model 4A has additive effects only and uses 41,023 autosomal SNPs after removing the 85 significant SNPs. $\widehat{\text{R}}_{{0{\text{jp}}}}$ is the observed accuracy of predicting phenotypic values from tenfold validations. ${\text{R}}_{{0{\text{jp}}}}$ is the expected accuracy of predicting phenotypic values. ${\text{R}}_{{0{\text{j}}}}$ is the expected accuracy of predicting genetic values calculated by GVCBLUP from tenfold validations, ${\text{j }} = {\text{t }}\;{\text{or}}\;\upalpha$. ${\text{h}}_{\text{t}}^{2}$ = 0.400 for Model 1A, = 0.297 for Model 1B, = 0.382 for Model 3. $\widehat{\text{h}}_{\upalpha}^{2}$ = 0.368 for Model 2A, 0.263 for Model 2B, = 0.350 for Model 4. $- {\text{c}}_{\text{ri}}$ is the decrease in accuracy

### GWAS results

**Fig. 3:**
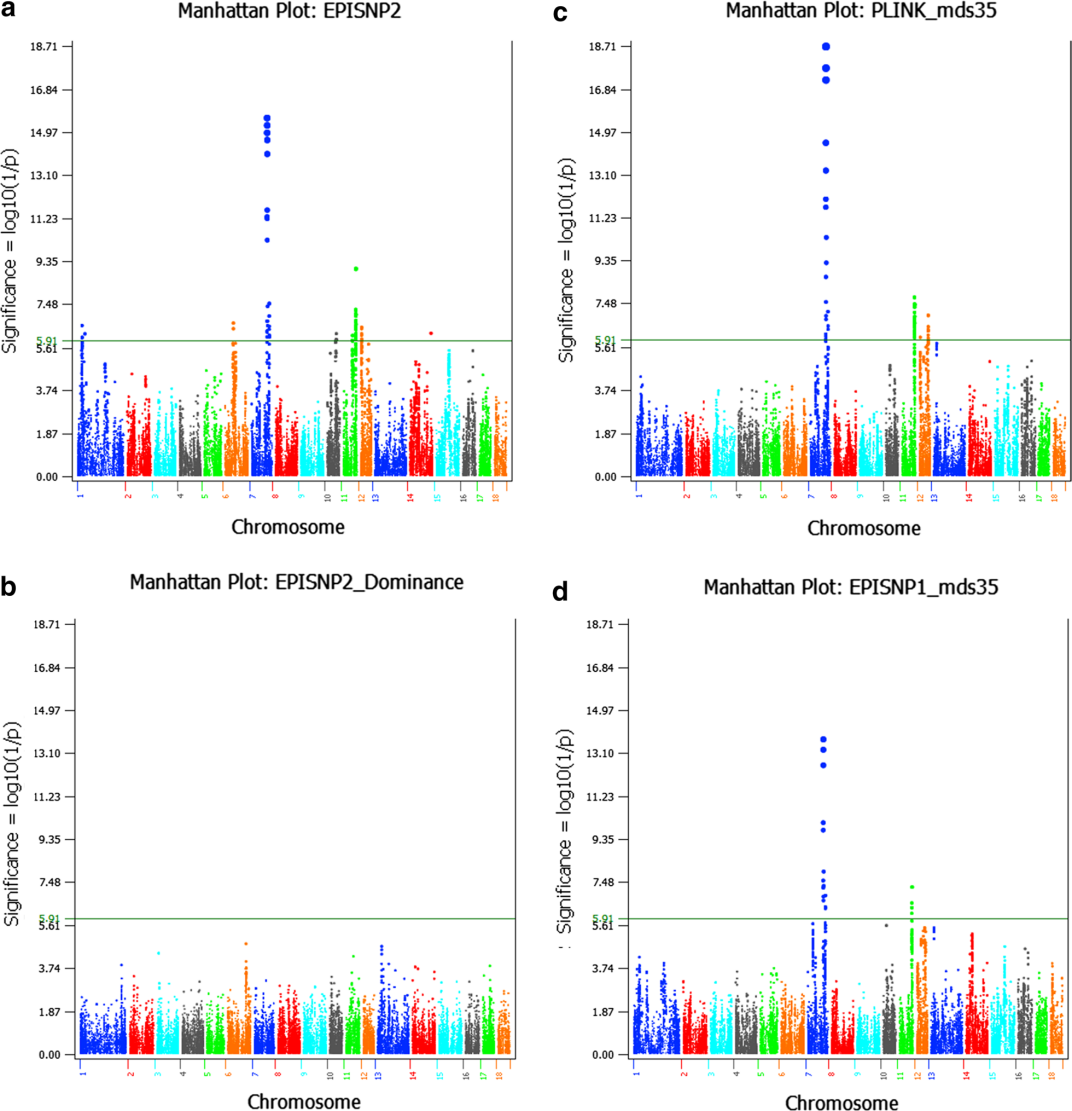
Manhattan plots from three methods of genome-wide association analysis. **a** Manhattan plot of p values for testing additive SNP effects using the generalized least squares (GLS) analysis of EPISNP2. **b** Manhattan plot of p values for testing dominance SNP effects using the generalized least squares (GLS) analysis of EPISNP2. **c** Manhattan plot of p values for testing additive SNP effects using the least squares (LS) analysis of PLINK with the first 35 dimensions of multidimensional scaling (MDS) as fixed effects. **d** Manhattan plot of p values for testing additive SNP effects using the LS analysis of EPISNP1 with the first 35 MDS dimensions as fixed effects. The *horizontal green line* indicates the genome-wide significance with the Bonferroni correction (p < 10^−5.91^). All p values in the figures are in log(1/p) scale

The GWAS that was done with EPISNP2, which accounted for the sib intraclass correlation and was implemented by a GLS analysis [11, 12], identified 73 SNPs on chromosomes 1, 6, 7, 10, 11, 12 and 14 with additive effects but no SNP with dominance effects reached genome-wide significance with the Bonferroni multiple testing correction (p < 10^−5.91^) (Fig. 3a, b; Table 3; Additional file 1: Table S1). LS analysis of PLINK [13] and EPISNP1 [11] with stratification correction using the first 35 dimensions of MDS as fixed covariates, identified 54 and 21 significant SNPs, respectively (Fig. 3c, d; Additional file 1: Table S1). Twelve SNPs detected by PLINK and two SNPs detected by EPISNP1 did not overlap with the SNPs detected by EPISNP2. Eighteen SNPs detected by EPISNP1 overlapped with those detected by EPISNP2 and PLINK. For this dataset, EPISNP1 was the most conservative for declaring significance. We report SNPs detected by EPISNP2 because they all had a substantial contribution to the broad-sense genomic heritability (see Additional file 1: Table S1). A graphical view of the GWAS results obtained by EPISNP2, PLINK and EPISNP1 for all autosomes is in Additional file 2: Figure S1.

**Table 3:** Chromosome regions with significant SNP effects on teat number

Chr	Region (Mb)	Size (Mb)	Most significant SNP				Contribution	Gene region
			Name	MAF	p value	% of ${\text{h}}_{\upalpha}^{2}$	% of $\widehat{\text{R}}_{0}$	
1	29.63–30.18	0.55	S1_29635241	0.499	2.68 (10^−07^)	2.25	1.51	*TNFAIP3, OLIG3*
7	102.91–103.80	0.89	S7_102911357	0.438	2.49 (10^−16^)	7.35	6.98	*PTGR2* (U), *FAM161B, LIN52*, *VRTN* (U, D), *FCF1*, *AREL1* (16 genes)
7	116.07–117.43	1.36	S7_116899295	0.342	2.97 (10^−08^)	3.07	2.13	*GALC*, *KCNK10*, *SPATA7*, *PTPN21*, *ZC3H14*, *EML5*, *TTC8*
11	56.56–58.58*	2.21	S11_58558301	0.422	7.14 (10^−7^)	1.64	2.27	*SPRY2*, *SNORA70*
11	77.75–79.69	1.94	S11_79009219	0.226	9.16 (10^−10^)	5.07	5.23	*FGF14*, *BIVM* (U, D), *LOC102167592, LOC102167785* (U)
12	4.53–6.26*	1.73	S12_5615207	0.335	3.16 (10^−07^)	2.59	2.63	*MFSD11*-*KCTD2* (49 genes)
12	50.54–51.74	1.20	S12_51574540	0.138	1.06 (10^−07^)	0.64	1.16	*OR3A2*, *ASPA*, *TRPV3*, *TRPV1*, *CTNS*, *TAX1BP3*, *EMC6*, *CAMKK1*

Chr = chromosome; MAF = minor allele frequency. ${\text{h}}_{\upalpha}^{2}$ is the additive heritability. $\widehat{\text{R}}_{0}$ is the observed accuracy of prediction. U indicate the significant SNP is located upstream of the gene. D indicates the significant SNP is located downstream of the gene. Contribution was calculated for the six most significant SNPs in each region. * This region has three significant SNPs

To evaluate the impact of the significant SNPs on the phenotypic variance, we estimated the decreases in observed genomic narrow-sense heritability and prediction accuracy when the phenotypic values were adjusted for the estimated genotypic values of the significant SNPs. The results showed that the 85 significant SNPs identified by the three methods, i.e. EPISNP2, PLINK and EPISNP1, accounted for 28.5 to 28.8% of the genomic narrow-sense heritability (Table 1) and for 36.2 to 36.8% of the observed prediction accuracy (Model 1A and Model 2A in Table 2). These results show that many SNPs that were deemed insignificant by the GWAS analysis were relevant for genomic prediction of teat number. Each of the 85 SNPs had a relatively large contribution to the genomic heritability and prediction accuracy, with the contribution of each SNP to the observed genomic narrow-sense heritability ranging from 0.7 to 7.3% and relative contribution of each SNP to the observed prediction accuracy ranging from 0.5 to 5.6% (see Additional file 1: Table S1).

### Analysis of the region between 102.9 and 106.0 Mb on chromosome 7

**Fig. 4:**
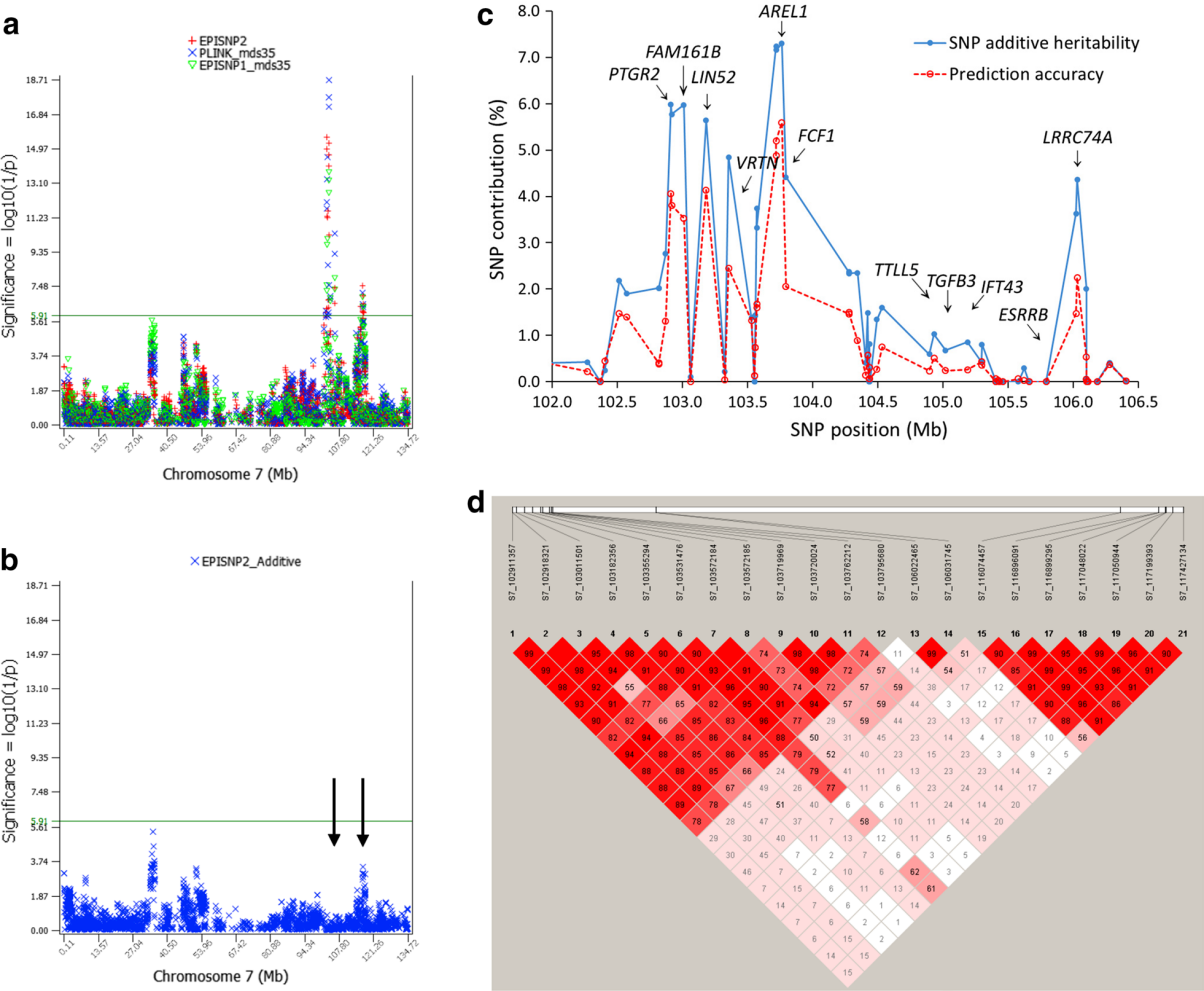
Analysis of the region between 102.9 and 106.0 Mb on chromosome 7. **a** Additive SNP effects by the generalized least squares analysis of EPISNP2 and by the least squares analysis of PLINK and EPISNP1, with stratification correction using the first 35 dimensions of multidimensional scaling. **b** Removal of the genotypic effects of the 14 SNPs with genome-wide significance by fitting these SNPs as fixed effects in the model completely removed all significant effects in this region and also removed the significant effects in the 116-Mb region on chromosome 7. **c** SNP contribution to genomic heritability and prediction accuracy of the 70 SNPs that are located within the region between 102.9 and 106.0 Mb, showing that the largest contributions originated from SNPs that were within or near the *AREL1* and *PTGR2* genes. **d** Linkage disequilibrium between the 21 significant SNPs in the region between 102.9 and 106.0 Mb on chromosome 7 by Haploview

A cluster of 14 SNPs within or near the *PTGR2*, *FAM161B*, *LIN52*, *VRTN*, *FCF1*, *AREL1* and *LRRC74A* genes in the region between 102.9 and 106.0 Mb on chromosome 7 had the most significant effects on teat number with genome-wide significance (Fig. 4a). Based on the GLS analysis of EPISNP2, the two SNPs upstream of *PTGR2* had the most significant additive effects, followed by the three SNPs within and upstream of *AREL1*, whereas the LS analysis of PLINK and EPISNP1 with stratification correction ranked the three *AREL1* SNPs as the most significant and the two SNPs upstream of *PTGR2* in the 6th and 7th positions (see Additional file 1: Table S1). The six most significant SNPs in the region between 102.9 and 103.8 Mb on chromosome 7 accounted for 7.4% of the genomic additive heritability and 7.0% of the observed prediction accuracy in the tenfold validation study (Table 3), and all the 14 SNPs in this region with genome-wide significance accounted for 10.0% of the genomic narrow-sense heritability and 8.0% of the observed prediction accuracy. Removal of the genotypic effects of the 14 SNPs by fitting these SNPs as fixed effects in the model for EPISNP2 removed all significant effects in the region between 102.9 and 103.8 Mb on chromosome 7 and also removed the significant effects of seven SNPs in the region between 116.1 and 117.4 Mb on chromosome 7 (Fig. 4b). SNPs within or near the *AREL1*, *PTGR2*, *FMA161B*, *LIN52* and *LRRC74A* genes also had the largest contributions to the genomic narrow-sense heritability and prediction accuracy (Fig. 4c). We did not detect SNPs within the *VRTN* gene but a SNP upstream of and nearest to *VRTN* (S7_103355294) was highly significant, ranking 6th based on EPISNP2 and 5th based on PLINK and EPISNP1. Linkage disequilibrium (LD) analysis using Haploview [20] showed that the two SNPs flanking *VRTN* were in strong LD (${\text{D}}^{\prime } = \, 0.90$, Fig. 4d), implying that either of these two SNPs could also be in strong LD with *VRTN*. Therefore, assuming that *VRTN* is a causal gene, the significant effect of S7_103355294 could be a linked effect of *VRTN*. The LD analysis showed that the significant effects of the region between 116.1 and 117.4 Mb on chromosome 7 could also be due to LD with the region between 102.9 and 106.0 Mb since three of the seven SNPs in the former region were in low LD with five significant SNPs in the latter region (${\text{D}}^{\prime } = \, 0.13\;{\text{to}}\;0.24$, Fig. 4d). This did not consider the possibility of multilocus LD between the two regions. The low LD was the only known reason that could explain the disappearance of the significant QTL effects of the region between 116.1 and 117.4 Mb when the 14 SNPs in the region between 102.9 and 106.0 Mb were fitted as fixed effects.

### Other chromosomes with significant SNPs

In addition to chromosome 7, SNPs on chromosomes 1, 6, 10, 11, 12 and 14 also had significant effects with genome-wide significance (Table 3; Additional file 1: Table S1). Among these chromosomes, the region between 77.3 and 79.7 Mb on chromosome 11 had the most significant additive effects. The top six SNPs within or near the *FGF14*, *BIVM*, *LOC10216759,* and *LOC102167785* genes accounted for 5.1% of the genomic narrow-sense heritability and 5.2% of the observed prediction accuracy (Table 3). The remaining regions on chromosomes 1, 7, 11 and 12 each with at least six SNPs accounted for 0.6 to 3.1% of the genomic narrow-sense heritability and accounted for 1.2 to 2.6% of the observed prediction accuracy (Table 3).

## Discussion

### Comparison with previous GWAS results

The region between 102.9 and 106.0 Mb on chromosome 7 that was identified in the current study was also reported in several previous GWAS but with varying lengths, e.g., between 102.1 and 105.2 Mb [2], 102.9 and 105.2 Mb [4], 103.0 and 103.6 Mb [3], the *VRTN* gene [5], the *VRTN*–*PROX2*–*FOS* region that is equivalent to the region between 103.4 and 104.3 Mb based on a microsatellite study [21], and the region between 106.7 and 106.9 Mb [7]. Five significant SNPs on chromosome 10 were located close to some previously reported significant regions on this chromosome [2], and a significant SNP at 51.6 Mb on chromosome 12 was close to the previously reported chromosome 12 region between 52.9 and 52.6 Mb [2, 4]. In the current study, the region between 77.7 and 79.7 Mb on chromosome 11 was the second most significant chromosome region, which, to our knowledge, has not been reported in previous GWAS. However, the Animal QTLdb [1] has one entry for pig teat number in the region between 79.2 and 85.7 Mb on chromosome 11, which partially overlaps the region between 77.7 and 79.7 Mb that we detected in this study, and this Animal QTLdb result was based on a QTL mapping study using 137 microsatellite markers on 573 F2 females and 530 F2 males from a Meishan × Large White cross [22].

### Heritabilities and factors that affect teat number

The genomic narrow-sense heritability estimates reported in the current study are the only ones available for teat number. Estimated narrow-sense heritabilities ranged from 0.346 to 0.350 (Table 1) and were within the range of recently published heritability estimates based on pedigree relationships, e.g., 0.39 in a study using 57,000 Yorkshire pigs [23], and 0.37 in a study using 1550 Landrace pigs [3]. The above genomic and pedigree-based heritability estimates indicate that swine teat number has a strong genetic component, and also that a large portion of the phenotypic variation is not explained by additive genetic effects. Only one GWAS reported dominance effects on chromosome 4 in Landrace pigs [3]. Our GWAS results differ from the reported dominance effects on chromosome 4 because we found that many other chromosomes had more significant dominance effects than chromosome 4, although none of the dominance effects that we observed reached the genome-wide significance threshold of p < 10^−5.91^ (Fig. 3b). The estimated dominance heritability was low (0.036) and inclusion of dominance effects in the prediction model only had negligible effects on the observed prediction accuracy. Removing dominance effects from the mixed model resulted only in a 0.5% reduction in the observed accuracy of predicting phenotypic values, although the reductions in the expected accuracy of predicting phenotypic values (7.6%) and in the expected accuracy of predicting genetic values (3.9%) were considerably larger for unknown reasons (Table 2). Previously, a maternal effect on teat number was reported [24] but has not been studied by GWAS or genomic prediction. Data error is a source of phenotypic variation, but teat number is easy to measure and any data errors that might have occurred would only be minor, given that our GWAS results on chromosome 7 agreed with those of other studies [2, 3] and that our genomic heritability estimates are consistent with those based on pedigree data [3, 23].

### SNP contributions to genomic heritability and prediction accuracy

We analyzed three methods (Methods I, II and III) of estimating the contribution of a set of SNPs to genomic heritability and compared Methods II and III for estimating the contributions of SNPs to both genomic heritability and prediction accuracy. Method I for estimating the contribution of SNPs to genomic heritability consisted of summing together the heritability estimates of the target set of SNPs because the narrow-sense, dominance and broad-sense heritabilities for each SNP can be estimated individually by the GVCBLUP package [18]. However, this method was inappropriate for estimating SNP contributions to the phenotypic variance because the heritability estimate for each SNP decreases as the number of SNPs in the mixed model increases and is approximately proportional to $1/{\text{m}}$, where ${\text{m}}$ is the number of SNPs in the mixed model (see “Appendix” for an approximate proof).

The results in Table 4 show the dependency of the size of the heritability estimate on the number of SNPs fitted as random effects in the mixed model. When dropping every other SNP from the model (i.e. reducing from 41,108 to 20,554 SNPs), the average ${\text{h}}_{{\upalpha{\text{i}}}}^{2}$ (narrow-sense heritability of the $i$th SNP) of each SNP for the same 20,554 SNPs when all 41,108 SNPs were fitted in the model nearly doubled (${\bar{\text{h}}}_{{\upalpha1}}^{2} /{\bar{\text{h}}}_{{\upalpha2}}^{2} = \, 1.960$), while the total narrow-sense heritability was nearly unaffected ($\widehat{\text{h}}_{{\upalpha1}}^{2} = \, 0.360$ and $\widehat{\text{h}}_{{\upalpha3}}^{2} = \, 0.368$), showing that the size of the heritability estimate for each SNP was approximately divided by 2 when the model had twice as many SNPs. Therefore, the heritabilities of the significant SNPs are not suitable for measuring their contributions to the phenotypic variance due to the dependency of the size of the heritability estimate for each SNP on the number of SNPs fitted as random effects in the mixed model.

**Table 4:** Estimates of SNP additive heritabilities of teat number when using 41,108 autosomal SNPs or every other (20,554) of the 41,108 SNPs

SNP set	Average $\widehat{\text{h}}_{{{\text{k}}\upalpha}}^{2}$ per SNP	Ratio	Total $\widehat{\text{h}}_{{\upalpha{\text{i}}}}^{2}$
20,554 SNPs	${\bar{\text{h}}}_{{\upalpha1}}^{2} = 1.75\;\left( {10^{ - 5} } \right)$	${\bar{\text{h}}}_{{\upalpha1}}^{2} /{\bar{\text{h}}}_{{\upalpha2}}^{2} = 1.960$	$\widehat{\text{h}}_{{\upalpha1}}^{2} = \, 0.360$
20,554 SNPs with all 41,108 SNPs in the mixed model	${\bar{\text{h}}}_{{\upalpha2}}^{2} = 8.93\;\left( {10^{ - 6} } \right)$	${\bar{\text{h}}}_{{\upalpha2}}^{2} /{\bar{\text{h}}}_{{\upalpha3}}^{2} = 0.998$	$\widehat{\text{h}}_{{\upalpha2}}^{2} = 0.184$
41,108 SNPs	${\bar{\text{h}}}_{{\upalpha3}}^{2} = 8.95\;\left( {10^{ - 6} } \right)$	${\bar{\text{h}}}_{{\upalpha1}}^{2} /{\bar{\text{h}}}_{{\upalpha3}}^{2} = 1.955$	$\widehat{\text{h}}_{{\upalpha3}}^{2} = \, 0.368$

$\widehat{\text{h}}_{{{\text{k}}\upalpha}}^{2}$ is the heritability of the ${\text{k}}$th SNP. ${\bar{\text{h}}}_{{\upalpha{\text{i}}}}^{2} = \sum\nolimits_{{{\text{k}} = 1}}^{\text{mi}} {\widehat{\text{h}}_{\text{ka}}^{2} } /{\text{m}}_{\text{i}}$ is the average of SNP additive heritability of the ${\text{i}}$th SNP set, $\widehat{\text{h}}_{{\upalpha{\text{i}}}}^{2} = \sum\nolimits_{{{\text{k}} = 1}}^{{{\text{m}}_{\text{i}} }} {\widehat{\text{h}}_{\text{ka}}^{2} }$ is the total additive heritability of all SNPs in the ${\text{i}}$th SNP set, where ${\text{m}}_{\text{i}}$ is the number of SNPs in the ${\text{i}}$th SNP set

Method II for estimating the contribution of a set of target SNPs to genomic heritability and prediction accuracy consisted in calculating the difference between the model with all SNPs and the model without the target SNPs. However, removing the target SNPs from the statistical model may not completely remove their effects because some of them could be explained by other SNPs in LD with the target SNPs. The results in Table 4 supported this expectation, i.e., the total narrow-sense heritability when halving the number of SNPs fitted was nearly unaffected. Using Method II, the 85 significant SNPs accounted for 4.5% of the total genomic heritability (Table 1) and 2.5% of the observed prediction accuracy (Model 3A, Table 2). Due to the partial effects of the removed SNPs that could have been explained by other SNPs, the contributions of SNPs to genomic heritability and observed prediction accuracy using Method II can be considered as the lower bound of the SNP contributions.

**Fig. 5:**
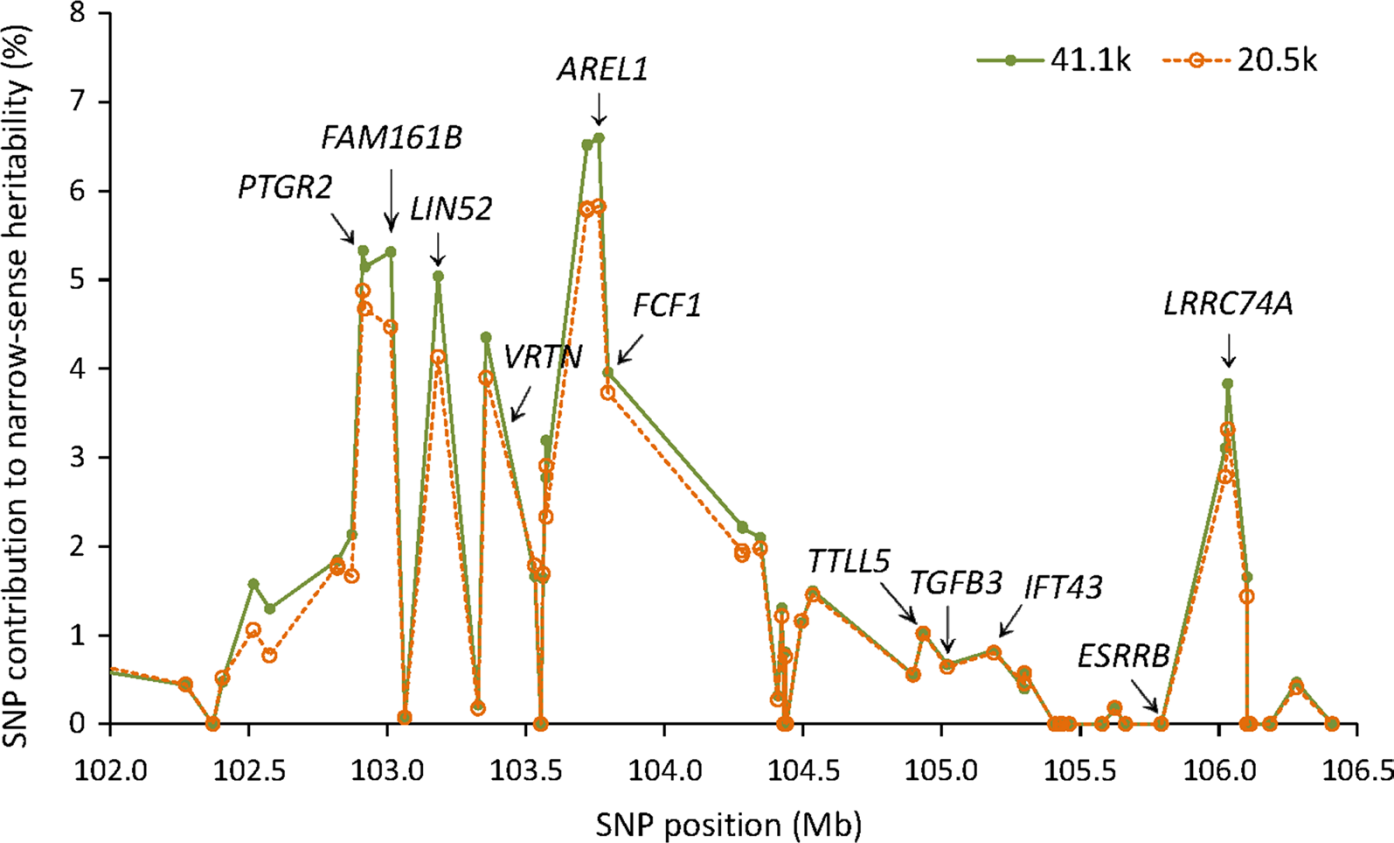
SNP partial heritability in the region between 102.9 and 106.0 Mb on chromosome 7 from two models with 20.5 and 41 K SNPs. The results show that partial heritability estimates were nearly unaffected by the number of SNPs in the model

Method III for estimating SNP contribution to genomic heritability and to prediction accuracy calculated the difference between the model with all SNPs fitted as random effects and the model with the target SNPs fitted as fixed effects, an approach that we refer to as ‘partial heritability’ and ‘partial accuracy’. The example of the region between 102.9 and 106.3 Mb on chromosome 7 showed that fitting significant SNPs as fixed effects completely removed the significant effects of those SNPs and also removed the effects of the SNPs that are still fitted in the statistical model as random effects but are in LD with the SNPs fitted as fixed effects (Fig. 4b). Figure 5 is a graphical view of a specific chromosome region, showing that the contributions of SNPs to the total narrow-sense heritability from the two models with 41,108 SNPs and 20,554 SNPs estimated using Method III were nearly the same for the region between 102.9 and 106.0 Mb on chromosome 7, i.e., partial heritability estimates were nearly unaffected by the number of SNPs in the model. Using this method, the 85 significant SNPs accounted for 28.5 to 28.8% of the genomic narrow-sense heritability (Table 1) and for 36.2 to 36.8% of the observed prediction accuracy (Table 2). In general, contributions of SNPs to genomic heritability and to the observed prediction accuracy were consistent, i.e., most SNPs with higher contributions to heritability also had greater contributions to prediction accuracies. On average, the contributions of SNPs obtained with Method III were larger than those with Method II by 24.0% (4.6 to 28.6%) for the genomic additive heritability and by 33.3% (2.3 to 35.6%) for the observed prediction accuracy (Table 2). Such large differences could be due to two reasons: overestimation by Method III and underestimation by Method II. Overestimation by Method III is expected since some effects that do not come from the target SNPs that are fitted as fixed effects could also be removed, in addition to removing the effects of the target SNPs. e.g., fitting the 14 SNPs in the region between 102.9 and 106.0 Mb on chromosome 7 as fixed effects also removed the significant effects of SNPs in the region between 116.1 and 117.4 Mb on chromosome 7 (Fig. 4b). Therefore, the contributions of SNPs estimated by Method III could be considered as the upper bound of the true SNP contributions. However, underestimation of Method II is likely the main reason for the large differences between Methods II and III, because a large percentage of the effects of the removed SNPs could have been explained by other SNPs in the model. For the sample in Table 4, the effects of half of the 41,108 SNPs were almost completely explained by the remaining half of the 41,108 SNPs because estimates of genomic heritabilities from those two sets of SNPs were almost the same, as we discussed above. Based on this analysis, we report contributions of SNPs by Method III in the abstract but also show the results obtained with Method II in the main body of the article (Tables 1, 2).

### Observed and expected prediction accuracies

In the current study, we compared three measures of prediction accuracy: the observed prediction accuracy of predicting phenotypic values based on the correlation between predictions from GBLUP and phenotypic observations of the validation individuals ($\widehat{\text{R}}_{{0{\text{jp}}}} ,\;{\text{j}} =\upalpha\;{\text{or}}\; {\text{t}}$), the expected accuracy of predicting phenotypic values (${\text{R}}_{{0{\text{jp}}}}$), and the expected accuracy of predicting genetic values (${\text{R}}_{{0{\text{j}}}}$). For the models using all 41,108 SNPs fitted as random effects (Model 1A and Model 2A in Table 2), we found excellent consistency between the observed accuracies of predicting phenotypic values ($\widehat{\text{R}}_{{0{\text{tp}}}} = \, 0.437$ for Model 1A and $\widehat{\text{R}}_{{0\upalpha{\text{p}}}} = \, 0.435$ for Model 2A) and the expected accuracies of predicting phenotypic values (${\text{R}}_{{0{\text{tp}}}} = \, 0.460$ for Model 1A and ${\text{R}}_{{0\upalpha{\text{p}}}} = \, 0.425$ for Model 2A). For the models using the 85 SNPs as fixed effects to remove the genetic values of those SNPs from the phenotypic values (Model 1B and Model 2B in Table 2), some differences between the observed accuracies of predicting phenotypic values ($\widehat{\text{R}}_{{0{\text{tp}}}} = 0.279$ for Model 1B and $\widehat{\text{R}}_{{0\upalpha{\text{p}}}} = 0.275$ for Model 2B) and between the expected accuracies of predicting phenotypic values (${\text{R}}_{{0{\text{tp}}}} = \, 0.360$ for Model 1B and ${\text{R}}_{{0\upalpha{\text{p}}}} = \, 0.320$ for Model 2B) were larger (Table 2), but those differences were mostly within one standard deviation of the observed accuracies and should be considered as acceptable. As expected, both observed and expected accuracies of predicting phenotypic values ($\widehat{\text{R}}_{{0{\text{jp}}}} \;{\text{and}}\;{\text{R}}_{{0{\text{jp}}}}$) were lower than the expected accuracies of predicting genetic values (${\text{R}}_{{0{\text{j}}}}$).

## Conclusions

Swine teat number has a strong genetic component with narrow-sense heritability estimates of about 0.365. The GWAS results confirmed the previously reported region on chromosome 7 and identified several new regions associated with swine teat number; they also indicated that the additive effects are the primary genetic effects for teat number and indicated consistency between statistical significance of SNP effects and SNP contribution to the genomic heritability. Most SNPs with higher statistical significance also had greater contributions to the genomic broad-sense heritability and prediction accuracy. The 85 significant SNPs accounted for about 28% of the genomic heritability and 36% of the prediction accuracy.

### Additional files

**Additional file 1: Table S1.** Significant SNP effects with Bonferroni significance (p < 10^−5.91^) by three methods of GWAS analysis.

**Additional file 2: Figure S1.** Manhattan plots of additive SNP effects of all 18 autosomes by three methods of GWAS analysis. All p-values in the figures are in log(1/p) scale.

## Appendix: Approximate proof for the decrease in SNP heritability as the number of SNPs increases

The heritability estimate for each SNP decreases as the number of SNPs in the mixed model increases and is approximately proportional to $1/{\text{m}}$, where ${\text{m}}$ is the number of SNPs in the mixed model. An approximate mathematical proof for this result can be derived based on the invariance property of GBLUP and GREML to duplicating SNPs. Assuming a set of ${\text{m}}$ SNPs is duplicated ${\text{r}}$ times in the mixed model, GBLUP of genetic values (additive, dominance and genotypic values) of individuals and SNP genetic variance components, as well as the associated variance estimates by GREML are invariant to the duplication of SNPs, and GBLUP of SNP additive, dominance and genotypic effects differ from those without duplicate SNPs by the square root of ${\text{r}}$ [25]. In the example of additive SNP effects, $\widehat{\upalpha}_{\text{ri}} = \widehat{\upalpha}_{\text{i}} /\sqrt {\text{r}}$, where $\widehat{\upalpha}_{\text{ri}}$ is the additive GBLUP estimate of the $i$th SNP from the mixed model with ${\text{m}}$ SNPs repeated ${\text{r}}$ times, $\widehat{\upalpha}_{\text{i}}$ is the additive GBLUP estimate of the $i$th SNP from the model with ${\text{m}}$ SNPs. The additive heritability for the $i$th SNP from the mixed model with ${\text{m}}$ SNPs (${\text{h}}_{{\upalpha{\text{i}}}}^{2}$) is: ${\text{h}}_{{\upalpha{\text{i}}}}^{2} = \left( {\widehat{\upalpha}_{\text{i}}^{2} /\sum\nolimits_{{{\text{i}} = 1}}^{\text{m}} {\widehat{\upalpha}_{\text{i}}^{2} } } \right){\text{h}}_{\upalpha}^{2}$ [18], where $\widehat{\upalpha}_{\text{i}}$ is the additive GBLUP of the $i$th SNP and ${\text{h}}_{\upalpha}^{2}$ is the total additive heritability based on all SNPs. Since ${\text{h}}_{\upalpha}^{2}$ is unaffected by repeated SNPs, the additive heritability for the $i$th SNP from the mixed model with ${\text{m}}$ SNPs repeated ${\text{r}}$ times (${\text{h}}_{{\upalpha{\text{ri}}}}^{2}$) is:

$${\text{h}}_{{\upalpha{\text{ri}}}}^{2} = \left( {{{\widehat{\upalpha}_{\text{ri}}^{2} } \mathord{\left/ {\vphantom {{\widehat{\upalpha}_{\text{ri}}^{2} } {\mathop \sum \limits_{{{\text{i}} = 1}}^{\text{rm}} \widehat{\upalpha}_{\text{ri}}^{2} }}} \right. \kern-0pt} {\mathop \sum \limits_{{{\text{i}} = 1}}^{\text{rm}} \widehat{\upalpha}_{\text{ri}}^{2} }}} \right){\text{h}}_{\upalpha}^{2} = {{\left( {\widehat{\upalpha}_{\text{i}}^{2} /{\text{r}}} \right)} \mathord{\left/ {\vphantom {{\left( {\widehat{\upalpha}_{\text{i}}^{2} /{\text{r}}} \right)} {\left( {\mathop \sum \limits_{{{\text{i}} = 1}}^{\text{rm}} \widehat{\upalpha}_{\text{i}}^{2} /{\text{r}}} \right)}}} \right. \kern-0pt} {\left( {\mathop \sum \limits_{{{\text{i}} = 1}}^{\text{rm}} \widehat{\upalpha}_{\text{i}}^{2} /{\text{r}}} \right)}}{\text{h}}_{\upalpha}^{2} = {\text{h}}_{{\upalpha{\text{i}}}}^{2} /{\text{r,}}$$

i.e., the heritability for the $i$th SNP from the model with ${\text{r}}$ times of the ${\text{m}}$ SNPs is $1/{\text{r}}$ of the SNP heritability with ${\text{m}}$ SNPs in the model without repeat. This theoretical result under the simple assumption of repeated SNPs was almost the same as the results obtained with the real data in Table 4, i.e., when the number of SNPs is reduced by half, the heritability of each SNP as an average of all SNP heritability estimates nearly doubled.
