# Supplementary figures and images for: Genome-wide association study and accuracy of genomic prediction for teat number in Duroc pigs using genotyping-by-sequencing
Source: Genet Sel Evol. 2017 Mar 29;49:35. doi: 10.1186/s12711-017-0311-8 (PMC5371258; doi:10.1186/s12711-017-0311-8)

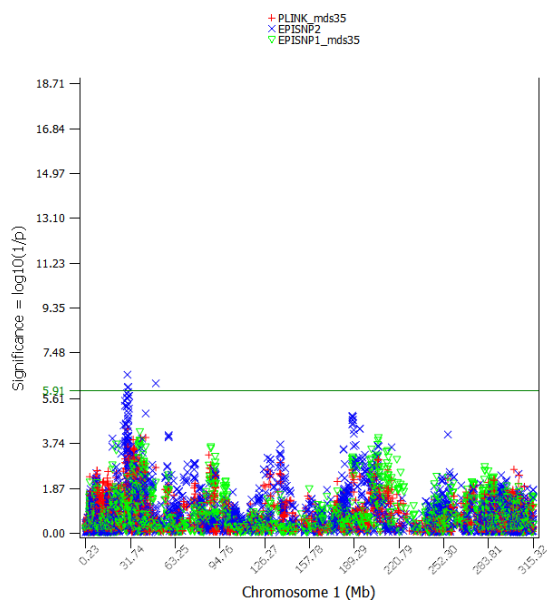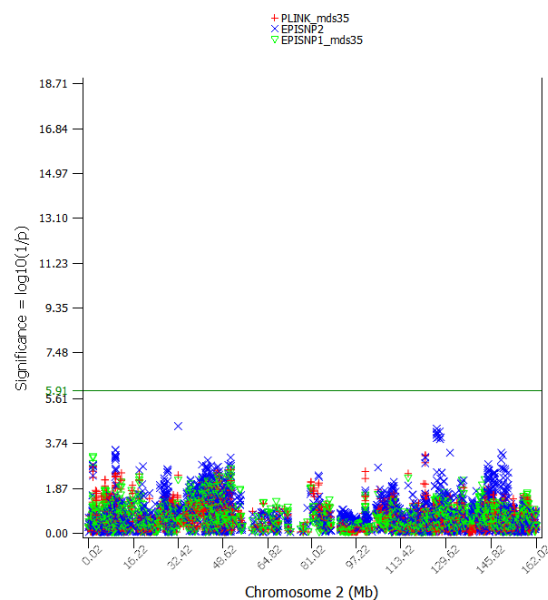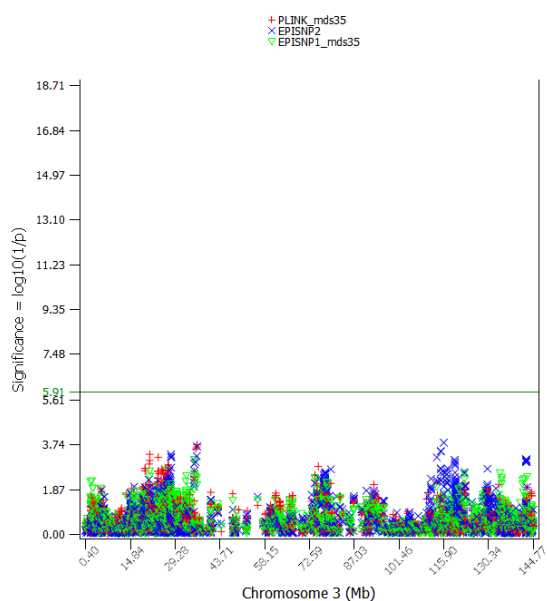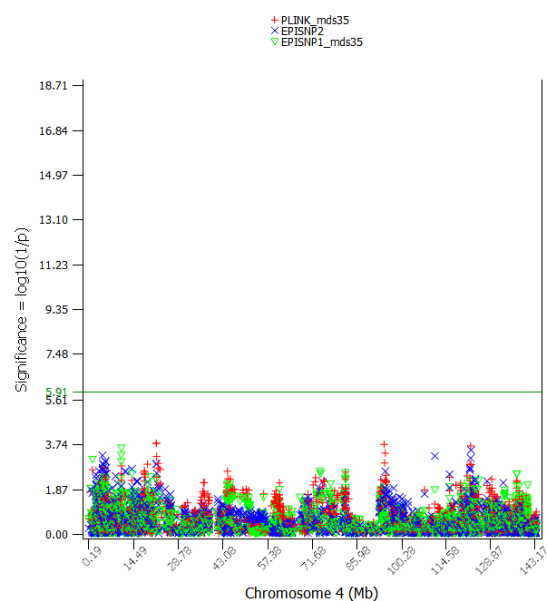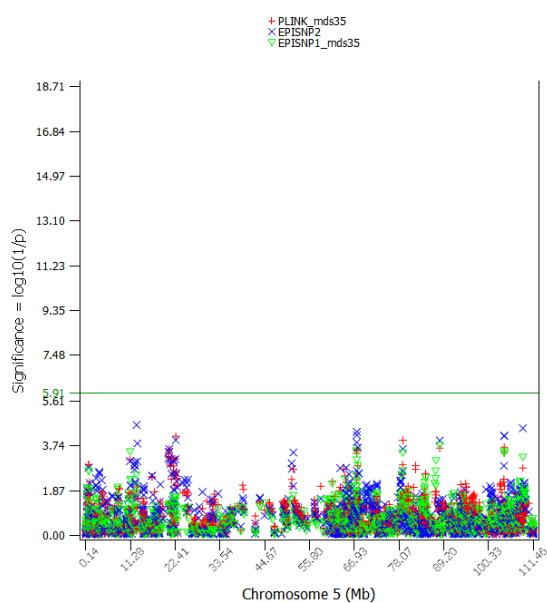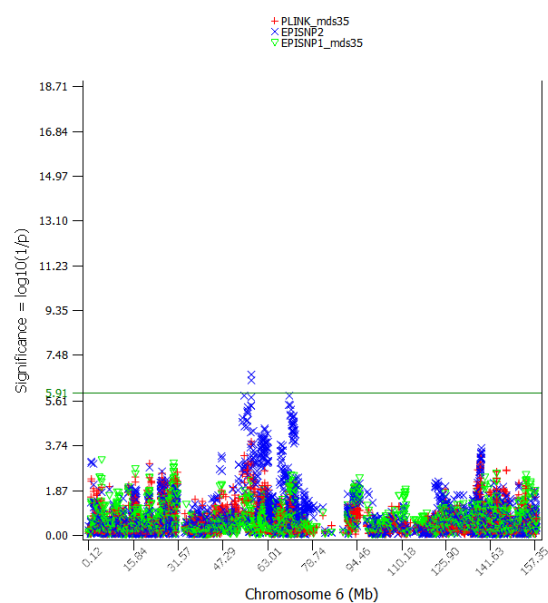

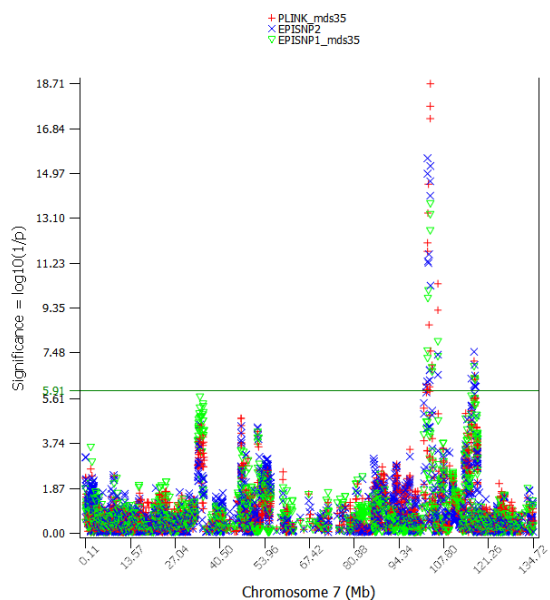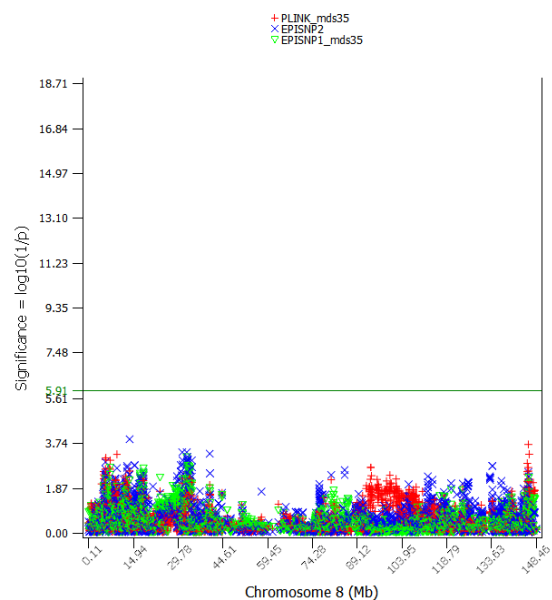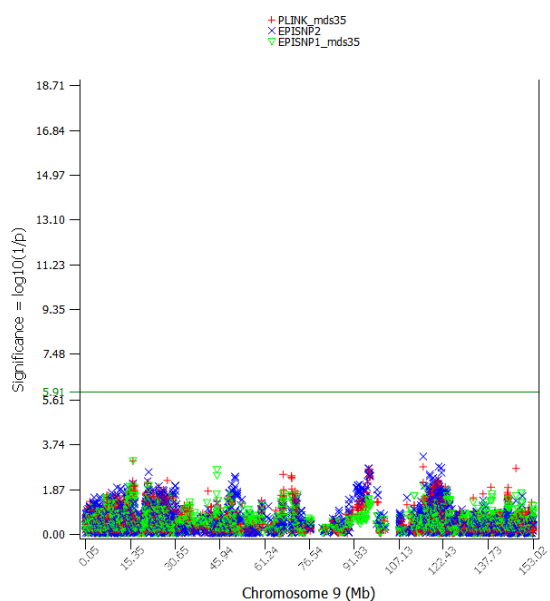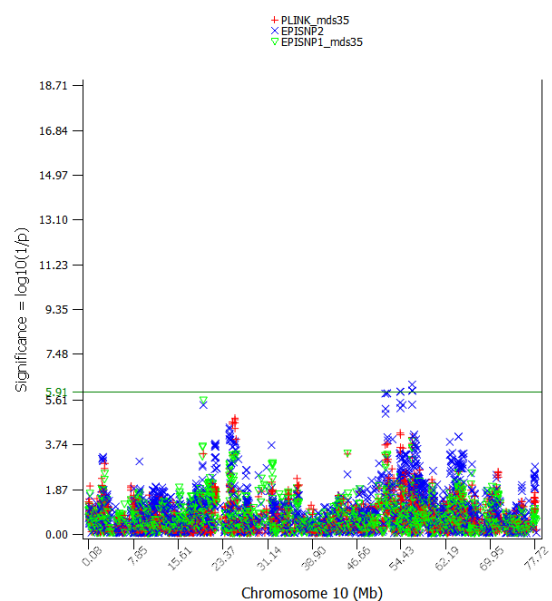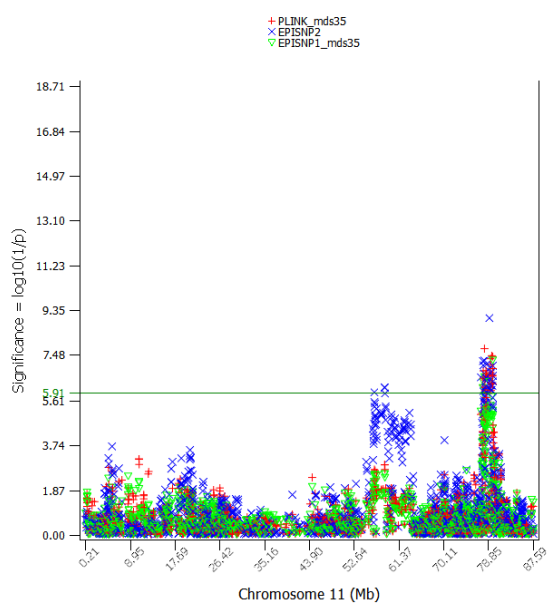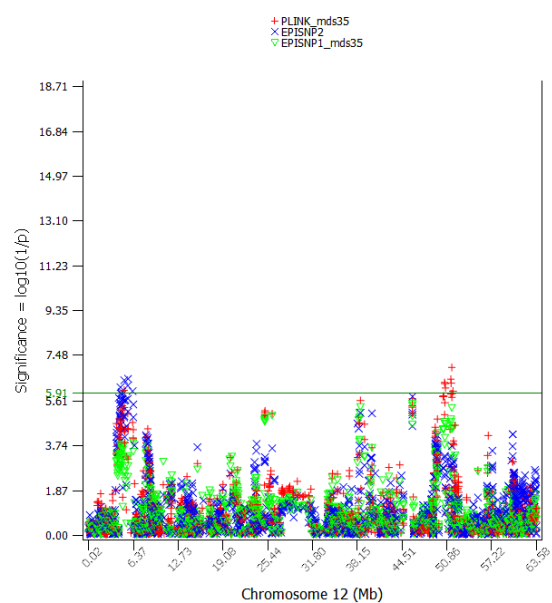

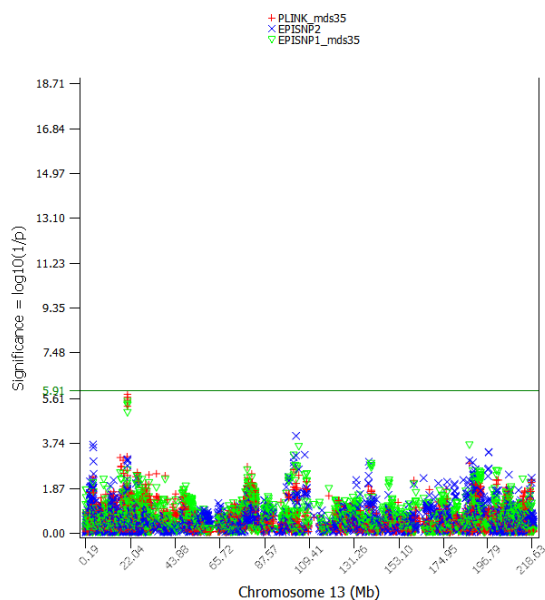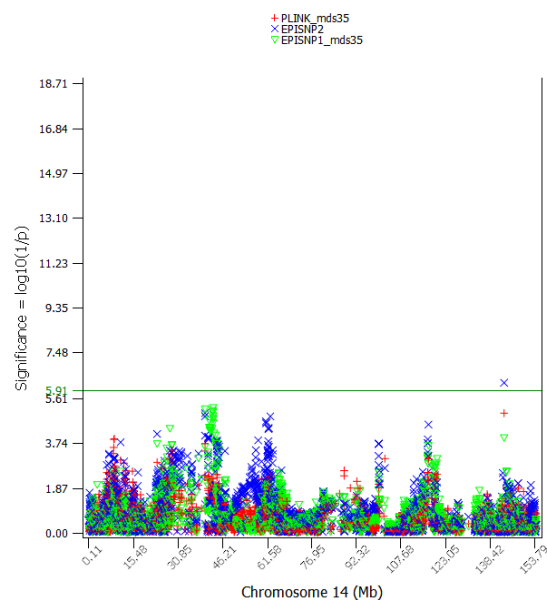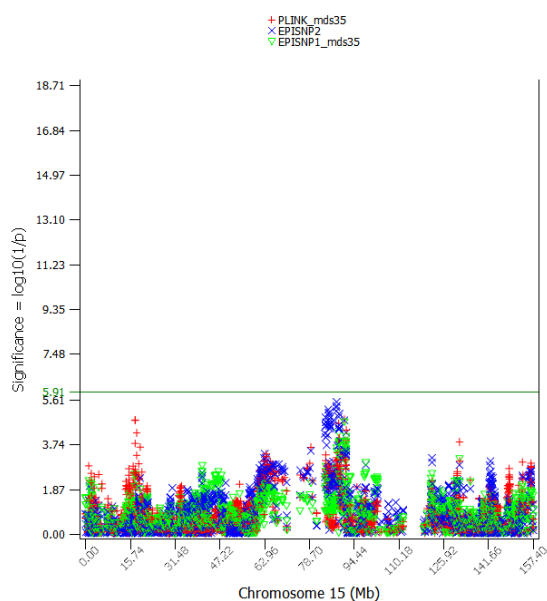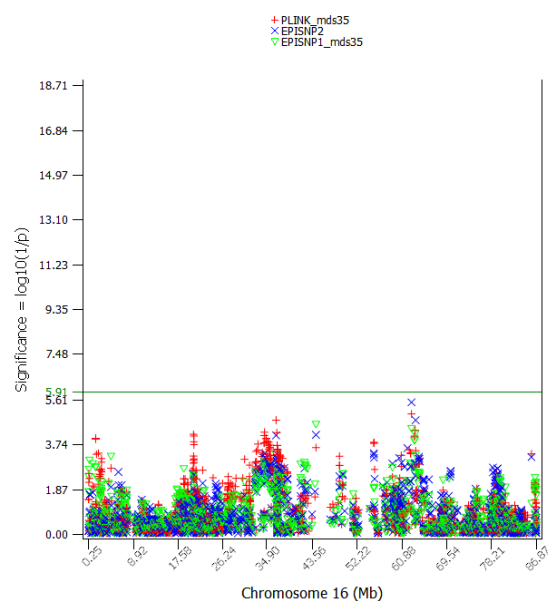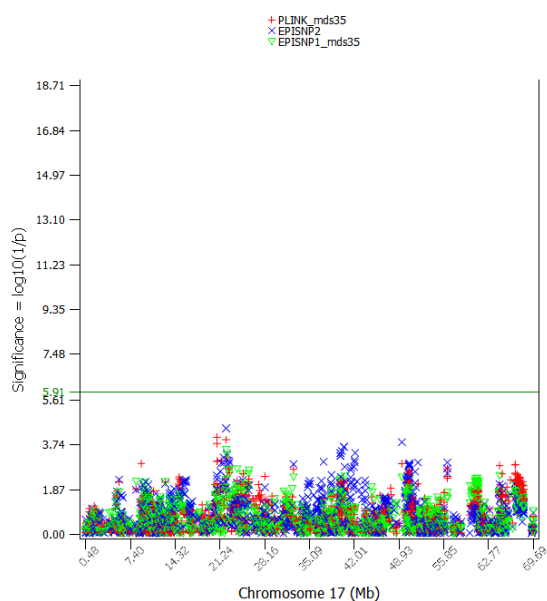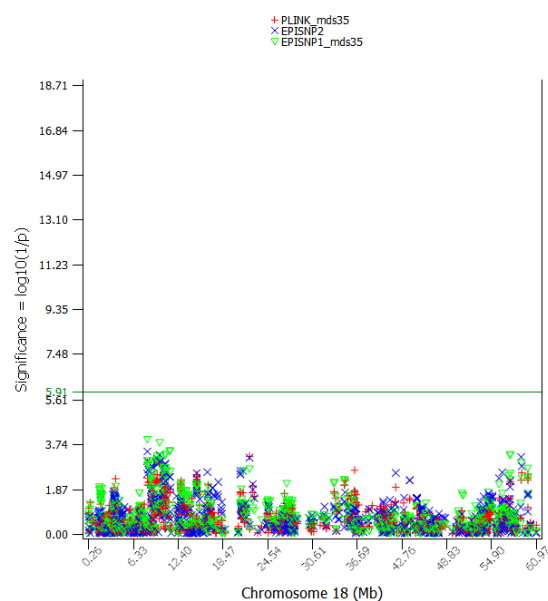

Supplement: Supplementary file 2 — Additional file 2: Figure S1. Manhattan plots of additive SNP effects of all 18 autosomes by three methods of GWAS analysis. All p-values in the figures are in log(1/p) scale. [file 12711_2017_311_MOESM2_ESM.pdf]
